# Serotype Distribution, Antimicrobial Susceptibility, Multilocus Sequencing Type and Virulence of Invasive *Streptococcus pneumoniae* in China: A Six-Year Multicenter Study

**DOI:** 10.3389/fmicb.2021.798750

**Published:** 2022-01-13

**Authors:** Menglan Zhou, Ziran Wang, Li Zhang, Timothy Kudinha, Haoran An, Chenyun Qian, Bin Jiang, Yao Wang, Yingchun Xu, Zhengyin Liu, Hong Zhang, Jingren Zhang

**Affiliations:** ^1^State Key Laboratory of Complex Severe and Rare Diseases, Department of Clinical Laboratory, Peking Union Medical College Hospital, Chinese Academy of Medical Sciences and Peking Union Medical College, Beijing, China; ^2^Beijing Key Laboratory for Mechanisms Research and Precision Diagnosis of Invasive Fungal Diseases, Beijing, China; ^3^Department of Infectious Disease, Peking Union Medical College Hospital, Chinese Academy of Medical Sciences, Beijing, China; ^4^School of Biomedical Sciences, Charles Sturt University, Orange, NSW, Australia; ^5^NSW Health Pathology, Regional and Rural, Orange Hospital, Orange, NSW, Australia; ^6^Department of Basic Medical Science, School of Medicine, Tsinghua University, Beijing, China; ^7^Tsinghua-Peking Center for Life Sciences, Tsinghua University, Beijing, China; ^8^Department of Clinical Laboratory, Hunan Provincial People’s Hospital, The First Affiliated Hospital of Hunan Normal University, Changsha, China; ^9^Department of Clinical Laboratory, Shanghai Children’s Hospital, Shanghai Jiao Tong University, Shanghai, China

**Keywords:** *Streptococcus pneumoniae*, serotype distribution, molecular epidemiology, antimicrobial susceptibility, virulence

## Abstract

**Background:** *Streptococcus pneumoniae* is an important human pathogen that can cause severe invasive pneumococcal diseases (IPDs). The aim of this multicenter study was to investigate the serotype and sequence type (ST) distribution, antimicrobial susceptibility, and virulence of *S. pneumoniae* strains causing IPD in China.

**Methods:** A total of 300 invasive *S. pneumoniae* isolates were included in this study. The serotype, ST, and antimicrobial susceptibility of the strains, were determined by the Quellung reaction, multi-locus sequence typing (MLST) and broth microdilution method, respectively. The virulence level of the strains in the most prevalent serotypes was evaluated by a mouse sepsis model, and the expression level of well-known virulence genes was measured by RT-PCR.

**Results:** The most common serotypes in this study were 23F, 19A, 19F, 3, and 14. The serotype coverages of PCV7, PCV10, PCV13, and PPV23 vaccines on the strain collection were 42.3, 45.3, 73.3 and 79.3%, respectively. The most common STs were ST320, ST81, ST271, ST876, and ST3173. All strains were susceptible to ertapenem, levofloxacin, moxifloxacin, linezolid, and vancomycin, but a very high proportion (>95%) was resistant to macrolides and clindamycin. Based on the oral, meningitis and non-meningitis breakpoints, penicillin non-susceptible *Streptococcus pneumoniae* (PNSP) accounted for 67.7, 67.7 and 4.3% of the isolates, respectively. Serotype 3 strains were characterized by high virulence levels and low antimicrobial-resistance rates, while strains of serotypes 23F, 19F, 19A, and 14, exhibited low virulence and high resistance rates to antibiotics. Capsular polysaccharide and non-capsular virulence factors were collectively responsible for the virulence diversity of *S. pneumoniae* strains.

**Conclusion:** Our study provides a comprehensive insight into the epidemiology and virulence diversity of *S. pneumoniae* strains causing IPD in China.

## Introduction

*Streptococcus pneumoniae* is a common Gram-positive coccus that can cause serious invasive pneumococcal diseases (IPDs) such as pneumonia, meningitis, and sepsis, especially in children and the elderly. It is estimated that IPD is responsible for approximately 826,000 deaths in children aged 1–59 months annually worldwide (O’Brien et al., 2009). Furthermore, the incidence of IPD increases with age, and with higher mortality reported in people over 65 years of age (Marrie et al., 2018).

The capsular polysaccharides of *S. pneumoniae* play an important role in pneumococcal disease pathogenesis, and at least 100 serotypes have been identified based on the capsule synthesis locus (*cps*) gene differences (Ganaie et al., 2020). The use of vaccines targeting specific serotypes has significantly reduced the morbidity and mortality of pneumococcal disease. *S. pneumoniae* vaccines PPV23 (covering serotypes 1–5, 6B, 7F, 8, 9 N, 9V, 10A, 11A, 12F, 14, 15B, 17F, 18C, 19A, 19F, 20, 22F, 23F, and 33F), PCV7 (covering serotypes 4, 6B, 9 V, 14, 18C, 19F, and 23F), PCV10 (covering PCV7 serotypes plus serotypes 1, 5, and 7F) and PCV13 (covering PCV10 serotypes plus serotypes 3, 6A, and 19A) were commercially introduced in the United States of America (USA) as early as 1983, 2000, 2008, and 2009 (Kim et al., 2020). At present, developed countries in Europe and the United States have already included PCV and PPV vaccines in their national vaccination programs, greatly reducing IPD caused by serotypes covered by these vaccines (Richter et al., 2013; Esposito et al., 2014). In China, several vaccines such as PCV7, PCV13, and PPV23 are currently available, but vaccination rates are still low due to the high cost of the vaccines (Boulton et al., 2016; Liang et al., 2016; Chen et al., 2018). However, the use of vaccines results in a shift in *S. pneumoniae* serotype distribution, a phenomenon known as “serotype replacement”, in which the prevalence of serotypes covered in the vaccines decrease whilst that of serotypes not included in the vaccines increase (Geno et al., 2015). Besides, *S. pneumoniae* can undergo efficient intra- and interspecies DNA recombination, leading to changes in the capsule composition, molecular typing, antibiotic resistance, and virulence factors (Varghese et al., 2019). These phenomena present great challenges to the prevention and treatment of pneumococcal diseases.

There is still a scarcity of comprehensive studies on the epidemiological characteristics as well as the virulence levels of *S. pneumoniae* causing invasive infections in China. The aim of this multicenter study was to investigate the serotype and sequence type (ST) distribution, antimicrobial susceptibility, and virulence of *S. pneumoniae* isolates causing IPD in China.

## Materials and Methods

### Isolates Collection

A total of 300 invasive *S. pneumoniae* isolates from 27 teaching hospitals in 13 provinces in China, collected during the period January 2010–October 2015, were included in this study (Supplementary Figure 1). All isolates were non-replicate and obtained from sterile sites, with only the first isolate selected from the same patient. The majority (141/300, 47%) of the isolates were isolated from younger children (0 to 5 years), whilst 29 (9.7%) were from older children (5 to 18 years), 92 (30.7%) from adults aged 18 to 65 years, and 38 (12.7%) from patients >65 years old. The major specimen type was blood (72.7%, 218/300), followed by cerebrospinal fluid (CSF) (19.0%, 57/300), and then pleural effusion (5.7%, 17/300). Other specimen types included ascetic fluid (*n* = 4), joint tissue (*n* = 2), thoracic drainage fluid (*n* = 1) and lung tissue (*n* = 1). All isolates were identified as *S. pneumoniae* using Vitek MS system (BioMerieux, Rhône, France) and optochin susceptibility test.

### Serotyping

All the isolates were serotyped by the Quellung reaction, which was considered the gold standard for serotyping. Firstly, the *S. pneumoniae* serogroup/type was preliminarily determined by latex agglutination test using the checkerboard typing system (Lalitha et al., 1999). Then specific antiserum was mixed with the bacterial suspension and capsular swelling was observed under the microscope. If the Quellung reaction was negative with all antisera, it was classified as non-typeable (NT) type.

### Multi-Locus Sequence Typing

The DNA of the *S. pneumoniae* isolates was extracted using the AxyGenamp DNA Mini Extraction Kit (Axygen, United States) according to the manufacturer’s instructions. Using the extracted DNA as the template, the seven housekeeping genes (*aroE, gdh, gki, recP, spi, xpt, ddl*) were amplified using polymerase chain reaction (PCR). The primer sequences used are detailed in Supplementary Table 1. The amplified products were sequenced and aligned with the sequences on the MLST website to determine the sequence type (ST) of the isolate. If a new allele combination was obtained, the corresponding sequence and strain-related information were submitted to the MLST databases^1^ for assigning of a new ST accession number after approval.

### Antimicrobial Susceptibility Testing

The minimum inhibitory concentrations (MICs) of *S. pneumoniae* isolates against penicillin (P), amoxicillin/clavulanic (AMC), cefuroxime (CXM), ceftriaxone (CRO), and several other antibiotics (cefepime, ertapenem, imipenem, meropenem, levofloxacin, trimethoprim/sulfamethoxazole, clindamycin, clarithromycin, erythromycin, linezolid and vancomycin), were determined by broth microdilution method. *S. pneumoniae* ATCC 49619 and *Escherichia coli* ATCC25922 were used as quality control strains. Each batch of isolates was tested simultaneously with the quality control strains, and the results were considered valid when the MIC values of both strains were within the expected quality control range. The antimicrobial susceptibility testing results were interpreted according to CLSI, 2019 document (CLSI, 2019).

### Virulence Test *in vivo*

This study was approved by the Medical Ethics Committee of Peking Union Medical College Hospital (No. S-263). The strains from the most prevalent serotypes in this study, and all serogroup six strains, were tested for virulence using a mouse sepsis model. Overall, 65 *S. pneumoniae* isolates were tested in this model, including serotypes 23F (*n* = 8), 19F (*n* = 8), 19A (*n* = 8), 3 (*n* = 8), 14 (*n* = 8), 6A (*n* = 12), 6B (*n* = 11), and 6C (*n* = 2).

We established an intraperitoneal infection model for *S. pneumoniae* using 6-week-old outbred CD-1 female mice (obtained from Beijing Vital River Laboratory Animal Technology Co. Ltd). For each isolate tested, a group of six mice were inoculated intraperitoneally with 200 μl of bacterial suspension containing approximately 1 × 10^4^ CFU of *S. pneumoniae* per mouse. The mortality of the mice was assessed daily until the 7th day. Isoflurane (Sigma-Aldrich, United States) was used at a dose of 6 mg/kg of the mouse weight for anesthetization at the time of blood collection. Orbital vein blood samples were obtained every 12 h for the first 2 days after inoculation and then daily for the next 5 days. Quantification of bacteria in the blood was determined by serial dilution and plating on trypticase soy agar with 3% sheep blood.

### Analysis of Virulence Gene Expression

RNA from 25 strains of serogroup 6 was extracted using Magen RNA Extraction Kit (Magen Bio, China) according to manufacturer’s instructions. Reverse transcription of the RNA was performed using the FastKing RT Kit (Tiangen Bio, China). Quantitative PCR reactions were performed using the SYBR Premix ExTaq™ PCR kit (Takara Bio, Japan) on a LightCycler 480 instrument (Roche Molecular Diagnostics, Rotkreuz, Switzerland). Using 16S rRNA as an internal reference gene, the expression of six non-capsule-associated virulence genes (*ply, lytA, nanA, psaA, pspA, and HylA*) (Ogunniyi et al., 2002; LeMessurier et al., 2006) was calculated using the 2^–ΔΔ*Ct*^ method. The primer sequences used are detailed in Supplementary Table 1.

### Statistical Analysis

Data on the distribution of different serotypes and vaccine coverage was analysed using Excel 2019 software (Microsoft Inc., United States). Differences in antimicrobial susceptibility were analyzed by MIC range, MIC_50_ and MIC_90_, and statistical analysis was performed by chi-square test or Fisher’s exact probability test using SPSS software (version 22.0, SPSS Inc., Chicago, IL, United States). Differences in survival time and gene expression between and within groups were analyzed by independent sample *t*-test and one-way ANOVA. *P*-values < 0.05 were considered statistically significant.

## Results

### Serotype Distribution

**FIGURE 1:**
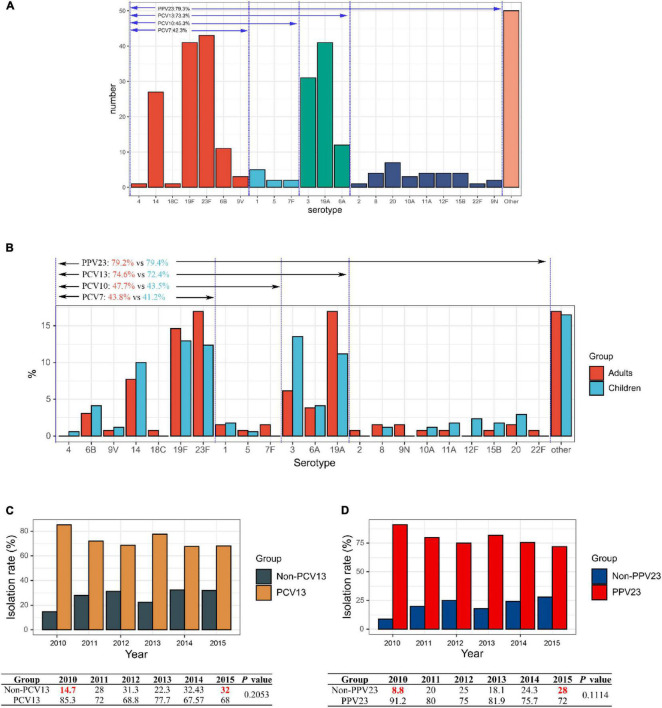
Serotype distribution of 300 invasive *S. pneumoniae* isolates. **(A)** Vaccine coverage *S. pneumoniae* isolates involved in this study. **(B)** Serotype distribution of isolates in different age groups in adult and children. **(C)** Changes in isolation rates of PCV13 and non-PCV13 covered serotypes strains during the study period 2010–2015. **(D)** Changes in isolation rates of PCV23 and non-PCV23 covered serotypes strains during the study period 2010–2015.

The serotypes of 299 *S. pneumoniae* strains were accurately identified by Quellung reaction, and the remaining one isolate was considered as non-typeable (NT). Overall, 40 serotypes were identified, with the 5 commonest being 23F (14.3%, 43/300), 19F (13.7%, 41/300), 19A (13.7%, 41/300), 3 (10.3%, 31/300), and 14 (9.0%, 27/300) (Supplementary Table 2). Based on the specific serotypes included in the vaccines, the coverage rates of PCV7, PCV10, PCV13, and PPV23 vaccines on this bacterial collection were 42.3% (127/300), 45.3% (136/300), 73.3% (220/300), and 79.3% (238/300), respectively (Figure 1A). The most prevalent serotypes isolated in the adult and children’s groups were similar. In the adult group, serotypes 23F (16.9%) and 19A (16.9%) were dominant, followed by 19F (14.6%), 14 (7.7%), and 3 (6.15%), whereas in the children group, serotype 3 was dominant (13.5%), followed by 19F (12.9%), 23F (12.4%), 19A (11.2%), and 14 (10.0%). There was no significant difference in the distribution of vaccine coverage between the adult and children’s groups: PCV7 (43.8% vs 41.2%) (*P* = 0.7386), PCV10 (47.7% vs 43.5%) (*P* = 0.5438), PCV13 (74.6% vs 72.4%) (*P* = 0.4224) and PPV23 (79.2% vs79.4%) (*P* = 0.9192) (Figure 1B).

Serotypes 23F, 19F, 19A, 3, and 14 were dominant from 2010 to 2015, ranging from 52.0 to 76.4%, albeit slight yearly variation in the distribution. In contrast, from the year 2010–2015, there was a decrease in the isolation rates of serotypes 23F, 19A, and 14, while that of 19F, 3, 6A, and 6B increased, although the differences were not statistically significant (Supplementary Figure 2). Noticeably, for both PCV13 and PPV23 vaccines, the vaccine serotype coverage among the isolates showed a decreasing trend from 2010 to 2015 (PCV13: 85.3 to 68%; PPV23: 91.2 to 72%), while the proportion of non-vaccine covered serotypes increased (non-PCV13: 14.7 to 32%; non-PPV23: 8.8 to 28%), though the difference was not statistically significant (*P* = 0.2053 for PCV13 and *P* = 0.1114 for PPV23) (Figures 1C,D).

### Multi-Locus Sequence Typing

Among the 300 isolates studied, 123 STs were identified by MLST analysis (Supplementary Table 3). Of these, ST320 (11.3%, 34/300) predominated, followed closely by ST81 (9.3%, 28/300), ST271 (8.7%, 26/300), ST876 (8.7%, 26/300) and ST3173 (2.7%, 8/300). There were 33 STs identified for the first time here, including ST12901-ST12920, ST13199-ST13200, ST14346-ST14351, ST14705-ST14707, and ST14726-ST14727.

**FIGURE 2:**
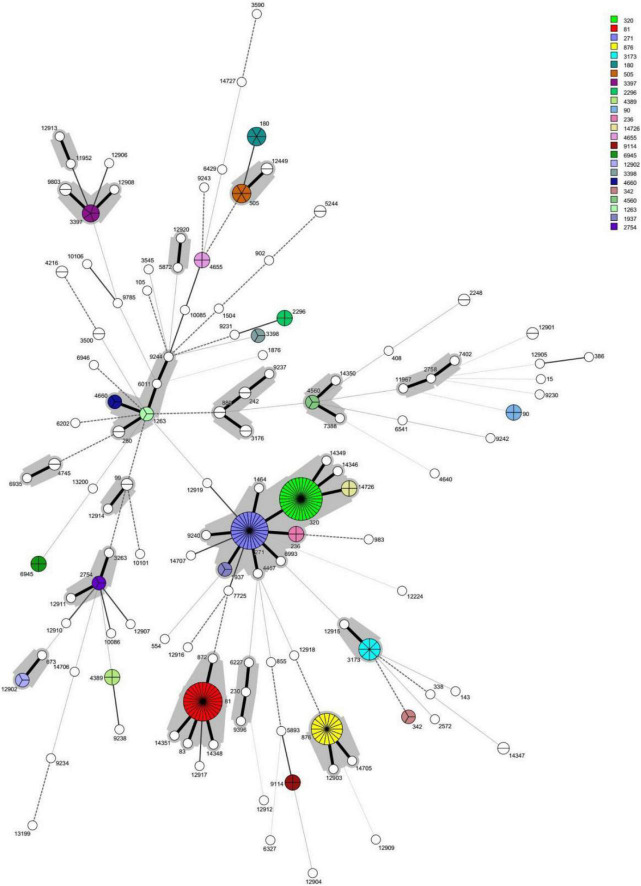
The minimun spanning tree (MST) of the 300 invasive *S. pneumoniae* isolates based on MLST. Note: Each circle corresponds to a sequence type (ST). The number besides the circle represents ST number. The circle size represents the number of strains. The circle color represents different ST categories with isolate number ≥3. The lines between circles indicate the similarity between profiles: bold solid line, six of seven MLST alleles in common; normal solid line, five alleles in common; dashed line, four alleles in common; dotted line, ≤3 alleles. The gray halo surrounding the STs in Figure denotes STs belonging to different MLST clusters/clonal complexes.

A phylogenetic tree was constructed based on single-locus variants (SLV) in seven housekeeping genes, and a total of 18 clonal complexes (CC) and 65 singletons were identified (Figure 2). The most common clonal complex was CC320 (13.3%), followed by CC271 (12.3%), CC81 (11.0%), CC876 (6.7%), and CC1263 (3.3%) (Table 1). Based on the Pneumococcal Molecular Epidemiology Network database (PMEN), seven international resistance clones containing 46 strains were identified in this study, including Spain^23*F*^-1 (ST81, *n* = 28), Netherlands^3^-31 (ST180, *n* = 6), Taiwan^19*F*^-14 (ST236, *n* = 4), Spain^6*B*^-2 (ST90, *n* = 4), Taiwan^23*F*^-15 (ST242, *n* = 2), Denmark^14^-32 (ST230, *n* = 1), and Colombia^23*F*^-26 (ST338, *n* = 1) (Supplementary Table 4).

**TABLE 1:** Summary of clonal complexes of 300 invasive *S. pneumoniae* isolates involved in this study.

CC/Singletons	No of isolates	Percentage (%)	Sequence types (STs)
CC320	40	13.3	ST320 (*n* = 34), ST14346 (*n* = 1), ST14349 (*n* = 1), ST14726 (*n* = 4)
CC271	37	12.3	ST271 (*n* = 26), ST236 (*n* = 4), ST1464 (*n* = 1), ST1937 (*n* = 3), ST4467 (*n* = 1), ST6993 (*n* = 1), ST9240 (*n* = 1)
CC81	33	11.0	ST81 (*n* = 28), ST83 (*n* = 1), ST872 (*n* = 1), ST12917 (*n* = 1), ST14348 (*n* = 1), ST14351 (*n* = 1)
CC876	20	6.7	ST876 (*n* = 18), ST12903 (*n* = 1), ST14705 (*n* = 1)
CC1263	10	3.3	ST280 (*n* = 2), ST1263 (*n* = 3), ST4660 (*n* = 3), ST6011 (*n* = 1), ST9244 (*n* = 1)
CC3173	9	3.0	ST3173 (*n* = 8), ST12915 (*n* = 1)
CC505	8	2.7	ST505 (*n* = 6), ST12449 (*n* = 2)
CC3397	8	2.7	ST3397 (*n* = 5), ST9803 (*n* = 2), ST12908 (*n* = 1)
CC880	7	2.3	ST880 (*n* = 2), ST242 (*n* = 2), ST3176 (*n* = 2), ST9237 (*n* = 1)
CC2754	5	1.7	ST2754 (*n* = 3), ST3263 (*n* = 1), ST12911 (*n* = 1)
CC4560	5	1.7	ST4560 (*n* = 3), ST7388 (*n* = 1), ST14350 (*n* = 1)
CC673	4	1.3	ST673 (*n* = 1), ST12902 (*n* = 3)
CC99	3	1.0	ST99 (*n* = 2), ST12914 (*n* = 1)
CC4745	3	1.0	ST4745 (*n* = 2), ST6935 (*n* = 1)
CC2758	3	1.0	ST2758 (*n* = 1), ST7402 (*n* = 1), ST11967 (*n* = 1)
CC230	3	1.0	ST230 (n = 1), ST6227 (*n* = 1), ST9396 (*n* = 1)
CC5872	2	0.7	ST5872 (*n* = 1), ST12920 (*n* = 1)
CC11952	2	0.7	ST11952 (*n* = 1), ST12913 (*n* = 1)
Singletons	98	32.7	ST180 (*n* = 6), ST4389 (*n* = 4), ST90 (*n* = 4), ST465 (*n* = 4)5, ST9114 (*n* = 4), ST2296 (*n* = 4), ST6945 (*n* = 4), ST3398 (*n* = 3), ST342 (*n* = 3), ST4216 (*n* = 2), ST12901 (*n* = 2), ST5244 (*n* = 2), ST2248 (*n* = 2), ST3500 (*n* = 2), ST14347 (*n* = 2), ST10106 (*n* = 1), ST9234 (*n* = 1), ST12910 (*n* = 1), ST143 (*n* = 1), ST9785 (*n* = 1), ST2572 (*n* = 1), ST12905 (*n* = 1), ST408 (*n* = 1), ST12919 (*n* = 1), ST902 (*n* = 1), ST9242 (*n* = 1), ST3545 (*n* = 1), ST10086 (*n* = 1), ST3590 (*n* = 1), ST1876 (*n* = 1), ST983 (*n* = 1), ST12907 (*n* = 1), ST105 (*n* = 1), ST12916 (*n* = 1), ST4640 (*n* = 1), ST855 (*n* = 1), ST338 (*n* = 1), ST9238 (*n* = 1), ST1504 (*n* = 1), ST9243 (*n* = 1), ST5893 (*n* = 1), ST10085 (*n* = 1), ST6202 (*n* = 1), ST10101 (*n* = 1), ST6327 (*n* = 1), ST12224 (*n* = 1), ST6429 (*n* = 1), ST12904 (*n* = 1), ST6541 (*n* = 1), ST12906 (*n* = 1), ST15 (*n* = 1), ST12909 (*n* = 1), ST6946 (*n* = 1), ST12912 (*n* = 1), ST13200 (*n* = 1), ST12918 (*n* = 1), ST14706 (*n* = 1), ST13199 (*n* = 1), ST14727 (*n* = 1), ST9231 (*n* = 1), ST7725 (*n* = 1), ST14707 (*n* = 1), ST386 (*n* = 1), ST554 (*n* = 1), ST9230 (*n* = 1)

In terms of annual distribution, ST320 dominated in 2010 (26.5%; 9/34), 2011 (12%, 3/25) and 2013 (12.8%, 12/94). Likewise, ST271 dominated in 2012, accounting for 12.5% (6/48) of the isolates, whilst ST81 (10.8%, 8/74), ST320 (12.0%, 3/25) and ST271 (12.0%, 3/25) dominated in 2014 and 2015, respectively. A declining trend in the prevalence of ST320 (from 26.5% in 2010 to 12% in 2015) and ST81 (from 14.7% in 2010 to 4% in 2015) was observed as can be seen in Supplementary Figure 3, whilst an increasing trend is obvious for ST271 (from 8.8% in 2010 to 12% in 2015) and ST876 (from 2.9% in 2010 to 10.8% in 2014) (Supplementary Figure 3).

**FIGURE 3:**
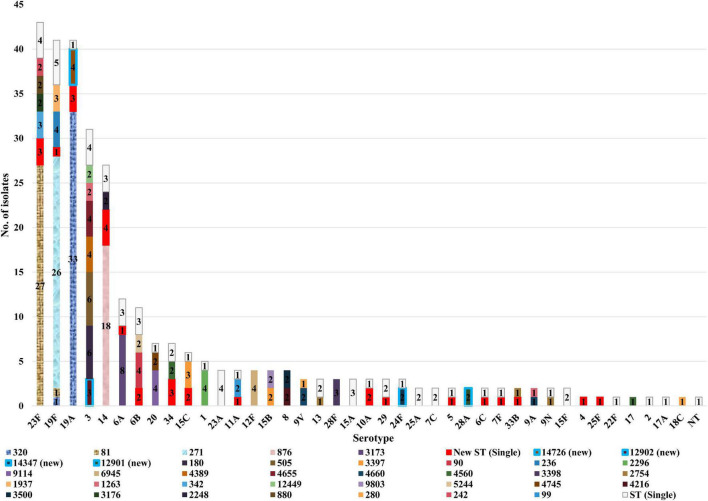
Sequence type distribution of 300 invasive *S. pneumoniae* isolates among different serotypes.

The distribution of STs among the different serotypes is shown in Figure 3. Only one ST was described in each of the following serotypes; 12F (*n* = 4), 28F (*n* = 3), 28A (*n* = 2), 4, 25F, 22F, 17, 2, 17A, and 18C (all *n* = 1, each). However, other serotypes showed diverse ST distribution. Serotype 23F was dominated by ST81 (63.0%, 27/43), whilst serotype 19F was dominated by ST271 (63.4%, 26/41) and serotypes 19A by ST320 (80.5%, 33/41). In addition, serotype 3 was dominated by ST505 (19.4%, 6/31) and ST180 (19.4%, 6/31), and serotype 14 by ST876 (66.7%, 18/27).

### Antimicrobial Susceptibility

Antimicrobial susceptibility results of the 300 invasive *S. pneumoniae* isolates are shown in Table 2. All the isolates were susceptible to ertapenem, levofloxacin, moxifloxacin, linezolid, and vancomycin, with MIC_90_ values of 0.25 μg/ml, 1 μg/ml, 0.125 μg/ml, 1 μg/ml and 0.5 μg/ml, respectively. This was closely followed by amoxicillin/clavulanic acid with a susceptible rate of 97.7% among the isolates. Over 90% of the strains were resistant to azithromycin, clarithromycin, erythromycin, clindamycin, tetracycline, and chloramphenicol. Notably, MIC_50_ and MIC_90_ values for azithromycin, clarithromycin and erythromycin were higher than 1024 μg/ml each. Based on the non-meningitis (*R* ≥ 8 μg/ml) breakpoint for penicillin, no resistant strains were observed among the isolates for this antibiotic, but only an intermediate rate of 4.3%. However, based on the meningitis (*R* ≥ 0.12 μg/ml) and oral (*R* ≥ 2 μg/ml) breakpoints for penicillin, the resistance rates for this antibiotic among the isolates were 67.7 and 44.7%, respectively. The resistance rate of the isolates to trimethoprim/sulfamethoxazole, cefuroxime and cefaclor was ≥60% each, with similar MIC_90_ values for ceftriaxone and cefepime at 2 μg/ml each. As per the meningitis breakpoint for cefepime, the resistance rate for cefepime was slightly higher than that of ceftriaxone (29.7% vs 25.7%), but slightly lower than that of ceftriaxone (4.0% vs 7.3%) when based on the non-meningitis breakpoint. A very small proportion of the isolates was resistant to imipenem (3.7%) and meropenem (2.7%), but the intermediate rate was higher at 37.7% (imipenem) and 46.0% (meropenem).

**TABLE 2:** Antimicrobial susceptibility results of the 300 *S. pneumoniae* isolates.

Antibiotics	*R* (%)	*I* (%)	*S* (%)	MIC_50_ (μg/ml)	MIC_90_ (μg/ml)	MIC range (μg/ml)
P*^a^*	0	4.3	95.7	1	2	≤0.015–4
P*^b^*	67.7	0	32.3	1	2	≤0.015–4
P*^c^*	44.7	23	32.3	1	2	≤0.015–4
AMC*^a^*	0.3	2	97.7	0.5	2	0.015–8
CXM*^d^*	64.3	2	33.7	4	16	0.06–64
CXM*^c^*	60	4.3	35.7	4	16	0.06–64
CRO*^a^*	7.3	18.3	74.3	0.5	2	0.007–8
CRO*^b^*	25.7	23.3	51	0.5	2	0.007–8
FEP*^a^*	4	25.7	70.3	1	2	0.03–8
FEP*^b^*	29.7	33	37.3	1	2	0.03–8
CEC	64	3	33	32	64	0.25–128
ETP	0	0	100	0.125	0.25	0.004–1
IPM	3.7	37.7	58.7	0.125	0.25	0.008–8
MEM	2.7	46	51.3	0.25	0.5	0.004–1
LEV	0	0	100	0.5	1	0.007–2
MXF	0	0	100	0.125	0.125	0.06–0.25
SXT	65.3	13	21.7	8	16	0.12–128
DA	95.7	1.7	2.7	128	128	0.12–256
AZM	96	0	4	>1024	>1024	0.25–2048
CLR	96	0	4	>1024	>1024	0.06–2048
E	96	0	4	>1024	>1024	0.06–2048
LZD	0	0	100	1	1	0.5–2
VA	0	0	100	0.5	0.5	0.015–1
C	91.7	0	8.3	8	16	0.25–64
TET	93.7	2.7	3.7	16	32	0.12–32

*^a^non-meningitis breakpoint.*

*^b^meningitis breakpoint.*

*^c^oral breakpoint.*

*^d^parenteral breakpoint.*

*P, penicillin; AMC, amoxicillin/clavulanic; CXM, cefuroxime; CRO, ceftriaxone; FEP, cefepime; CEC, cefaclor; ETP, ertapenem; IPM, imipenem; MEM, meropenem; LEV, levofloxacin; MXF, moxifloxacin; SXT, trimethoprim/sulfamethoxazole; DA, clindamycin; AZM, azithromycin; CLR, clarithromycin; E, erythromycin; LZD, linezolid; VA, vancomycin; C, chloramphenicol; TET, tetracycline.*

The susceptibility profiles of several predominant serotypes to different antimicrobial agents are shown in Table 3. Overall, serotypes 23F, 19F, 19A, and 14 exhibited a higher resistance rate to all the antimicrobials tested, while serotype 3 had a relatively lower resistance rate. All serotypes showed high resistance rates to macrolides (87.1–100%), chloramphenicol (87.8–100%) and tetracycline (83.9–100%). Furthermore, all serotypes except serotype 3, exhibited high resistance rates to penicillin (meningitis and oral breakpoint), cefuroxime (63.6–100%) and cefaclor (72.7–100%). Serotype 19F had higher resistance rates to ceftriaxone (39.0% based on the non-meningitis breakpoint and 75.6% based on the meningitis breakpoint) and cefepime (14.6% based on the non-meningitis breakpoint and 78.0% based on the meningitis breakpoint) compared to other serotypes.

**TABLE 3:** Antimicrobial susceptibility results of the 300 *S. pneumoniae* isolates with different serotypes in China.

Antibiotics	*R* (%)						
	23F (*n* = 43)	19F (*n* = 41)	19A (*n* = 41)	3 (*n* = 31)	14 (*n* = 27)	6A (*n* = 12)	6B (*n* = 11)
P*^a^*	0	0	0	0	0	0	0
P*^b^*	97.7	100	97.6	0	100	100	100
P*^c^*	81.4	70.7	97.6	0	77.8	66.7	0
AMC*^a^*	0	2.4	0	0	0	0	0
CXM*^d^*	86	97.6	100	9.7	92.6	83.3	63.6
CXM*^c^*	93	100	100	19.4	96.3	83.3	63.6
CRO*^a^*	4.7	39	7.3	0	0	0	0
CRO*^b^*	30.2	75.6	39	9.7	22.2	25	27.3
FEP*^a^*	4.7	14.6	4.9	3.2	0	0	0
FEP*^b^*	20.9	78	63.4	9.7	25.9	16.7	18.2
CEC	90.7	97.6	100	16.1	92.6	91.7	72.7
ETP	0	0	0	0	0	0	0
IPM	0	7.3	2.4	3.2	7.4	0	0
MEM	0	4.9	7.3	3.2	0	0	9.1
LEV	0	0	0	0	0	0	0
MXF	0	0	0	0	0	0	0
SXT	90.7	95.1	97.6	32.3	33.3	25	45.5
DA	100	100	100	87.1	100	100	100
AZM	100	100	100	87.1	100	100	100
CLR	100	100	100	87.1	100	100	100
E	100	100	100	87.1	100	100	100
LZD	0	0	0	0	0	0	0
VA	0	0	0	0	0	0	0
C	95.3	87.8	90.2	80.6	96.3	100	100
TET	100	97.6	100	83.9	85.2	100	100

*^a^non-meningitis breakpoint.*

*^b^meningitis breakpoint.*

*^c^oral breakpoint.*

*^d^parenteral breakpoint.*

*P, penicillin; AMC, amoxicillin/clavulanic; CXM, cefuroxime; CRO, ceftriaxone; FEP, cefepime; CEC, cefaclor; ETP, ertapenem; IPM, imipenem; MEM, meropenem; LEV, levofloxacin; MXF, moxifloxacin; SXT, trimethoprim/sulfamethoxazole; DA, clindamycin; AZM, azithromycin; CLR, clarithromycin; E, erythromycin; LZD, linezolid; VA, vancomycin; C, chloramphenicol; TET, tetracycline.*

Serotype 14 showed a high rate of resistance to imipenem (7.4%), serotype 6B showed a high rate of resistance to meropenem (9.1%), whereas serotype 6A exhibited a low level of resistance to trimethoprim/sulfamethoxazole (25.0%). We also observed that both PCV and PPV23 vaccine-covered serotype strains showed higher resistance rates to most antimicrobial drugs than the corresponding non-vaccine-covered serotype strains, including for penicillin, cefuroxime, ceftriaxone, cefepime (meningitis breakpoint), and cefaclor (Supplementary Table 5). In addition, a higher proportion of PCV-covered serotype strains were resistant to clindamycin than non-covered strains (97.3% vs 91.2%, *P* = 0.0482). Likewise, a higher percentage of PPV23-covered serotype strains were resistant to cotrimoxazole than non-covered serotype strains (68.9% vs 51.6%, *P* = 0.0164).

An analysis of overall resistance rates of the isolates to different antimicrobial drugs by year is shown in Table 4. Collectively, over the six-year study period, a decreasing trend in antibiotic resistance rates to penicillin, cefuroxime, ceftriaxone, cefepime, cefaclor and trimethoprim/sulfamethoxazole, was observed. However, the resistance rates for ertapenem, levofloxacin, moxifloxacin, linezolid, and vancomycin remained at zero. On the other hand, clindamycin, azithromycin, clarithromycin, erythromycin, chloramphenicol, and tetracycline, maintained high levels of resistance rates (81.2–100%) across the six-year period. The resistance rate of imipenem also underwent a fluctuation (2.9% in 2010, 0% in 2011 and 2012, and then 8.1 and 8.0% in 2014 and 2015) during the 6 years. For other antibiotics, such as amoxicillin/clavulanic acid and meropenem, resistant strains have emerged but the overall resistance rate was still no more than 5%.

**TABLE 4:** Antimicrobial susceptibility results of the 300 *S. pneumoniae* isolates during the study period in China.

Antibiotics	*R* (%)						*P*-value
	2010 (*n* = 34)	2011 (*n* = 25)	2012 (*n* = 48)	2013 (*n* = 94)	2014 (*n* = 74)	2015 (*n* = 25)	
P*^a^*	0	0	0	0	0	0	NA
P*^b^*	76.5	52	70.8	73.4	60.8	64	0.4491
P*^c^*	61.8	32	41.7	52.1	36.5	36	0.09
AMC*^a^*	0	0	0	0	1.4	0	NA
CXM*^d^*	67.6	52	60.4	66	51.4	60	0.7434
CXM*^c^*	73.5	52	66.7	72.3	51.4	68	0.865
CRO*^a^*	11.8	8	6.2	6.4	6.8	8	0.967
CRO*^b^*	44.1	16	29.2	25.5	14.9	36	0.7206
FEP*^a^*	8.8	4	2.1	4.3	4.1	0	0.3566
FEP*^b^*	52.9	24	33.3	24.5	23	36	0.3062
CEC	73.5	56	64.6	69.1	52.7	72	0.8664
ETP	0	0	0	0	0	0	NA
IPM	2.9	0	0	2.1	8.1	8	0.7778
MEM	0	0	4.2	4.3	2.7	0	NA
LEV	0	0	0	0	0	0	NA
MXF	0	0	0	0	0	0	NA
SXT	88.2	68	54.2	69.1	55.4	68	0.1148
DA	97.1	100	89.6	95.7	97.3	96	0.6179
AZM	100	100	87.5	95.7	98.6	96	0.8763
CLR	100	100	87.5	95.7	98.6	96	0.8763
E	100	100	87.5	95.7	98.6	96	0.8763
LZD	0	0	0	0	0	0	NA
VA	0	0	0	0	0	0	NA
C	100	88	89.6	88.3	94.6	92	0.3421
TET	100	92	81.2	97.9	93.2	96	0.8763

*^a^non-meningitis breakpoint.*

*^b^meningitis breakpoint.*

*^c^oral breakpoint.*

*^d^parenteral breakpoint.*

*NA, not available.*

*P, penicillin; AMC, amoxicillin/clavulanic; CXM, cefuroxime; CRO, ceftriaxone; FEP, cefepime; CEC, cefaclor; ETP, ertapenem; IPM, imipenem; MEM, meropenem; LEV, levofloxacin; MXF, moxifloxacin; SXT, trimethoprim/sulfamethoxazole; DA, clindamycin; AZM, azithromycin; CLR, clarithromycin; E, erythromycin; LZD, linezolid; VA, vancomycin; C, chloramphenicol; TET, tetracycline.*

### The Virulence Levels of Predominant Serotypes

**FIGURE 4:**
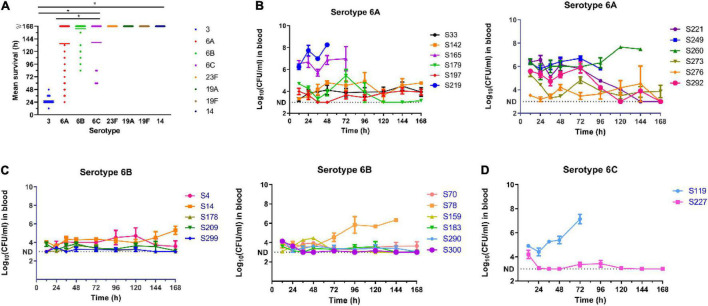
Virulence assessment of pneumococcal isolates in mouse sepsis model. **(A)** Relationship of the virulence levels and the serotypes of *S. pneumoniae*. Virulence level was determined by the mean survival time of mice i.p. infected with pneumococcal isolates. One-way ANOVA was used to compare differences among groups. **(B–D)** Blood burden of bacteria in mice infected with different pneumococcal strains in serotype 6A, 6B, and 6C. *P < 0.05.

The results of the virulence level assessment among the predominant serotype strains are shown in Figure 4A. We observed extremely low virulence levels for *S. pneumoniae* strains in serotypes 23F, 19A, 19F, and 14, with an average mice survival time uniformly exceeding 7 days. Conversely, 1 × 10^4^ CFU of serotype 3 strains killed all the mice within 2 days, with a mean survival time of 24 h. In addition, the number of bacteria in the blood of mice infected with serotype 3 strains (*n* = 8) increased rapidly to 10^5^ CFU/ml in the first 12 h of inoculation, and then increased up to 10^9^ CFU/ml at 24 h. For serotypes 23F, 19F, 19A, and 14 strains, the bacterial load remained undetectable over the course of the experiment. These results suggest that the virulence levels of *S. pneumoniae* isolates is somehow dependent on capsular serotypes. For instance, serotype 3 strains were highly virulent in the mouse model whilst serotypes 23F, 19A, 19F, and 14 were lowly virulent. However, for serogroup 6 strains, virulence level variation existed among the strains.

We defined strains with maximal bacterial load in the blood of ≥10^5^ CFU/ml and a 7-day survival rate ≤20% as high virulent strains (HVS), while those with bacterial blood load of ≤10^4^CFU/ml and a 7-day survival rate ≥80%, were defined as low virulence strains (LVS). The remainder was classified as intermediate virulent strains (IVS). Based on this criterion, six (S165, S219, S221, S249, S260, and S292) of the 12 strains of serotype 6A were HVS, two (S142 and S179) were LVS and the rest (S33, S197, S273, S276) IVS (Figure 4B). For serotype 6B, only one strain (S78) was classified as HVS, two (S4 and S14) as IVS and the remaining eight (S70, S159, S178, S183, S209, S290, S299, and S300) as LVS (Figure 4C). There were only two strains of serotype 6C, and one was classified as HVS (S119) and the other (S227) as LVS (Figure 4D).

**FIGURE 5:**
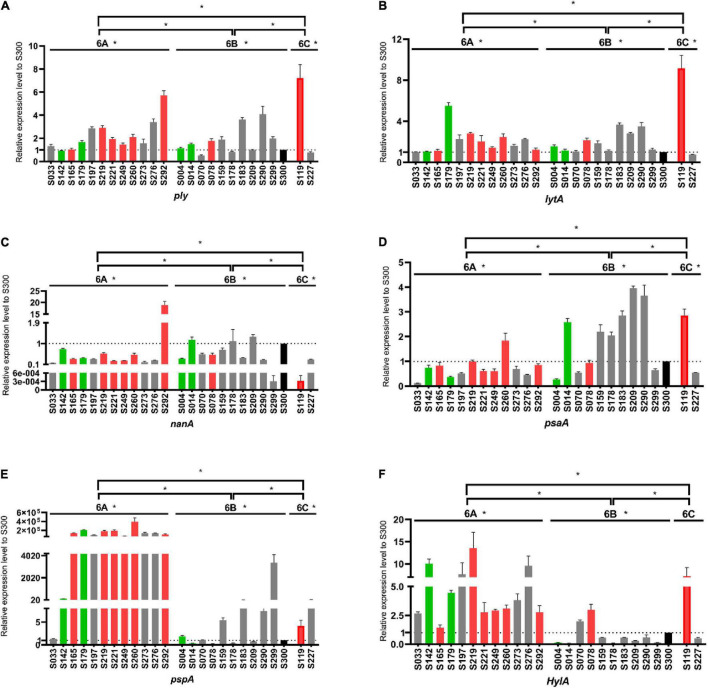
Comparison of virulence gene expression levels among serogroup 6 strains. Expression levels of *ply* **(A)**, *lytA* **(B)**, *nanA* **(C)**, *psaA* **(D)**, *pspA* **(E)** and *hylA* **(F)** were measured and compared with the S300 strain (marked in black) as the control strain. Gray, green and red represent the low virulence strains (LVS), intermediate virulent strains (IVS) and high virulent strains (HVS), respectively. *P < 0.05.

The virulence level variation among strains within the same serotype suggests that some capsule-independent factors may have contributed to virulence. Thus, the expression levels of six non-capsule-associated virulence genes (*ply*, *lytA*, *nanA*, *psaA*, *pspA*, and *hylA*) were measured by RT-PCR (Figure 5). In serotype 6A, strain S292 (HVS) had the highest-level expression of *ply* and *nanA* genes, whilst isolate S260 (HVS) had the highest level of expression of *psaA* and *pspA* genes. Furthermore, isolate S219 (HVS) had the highest level of expression of *hylA*, whilst isolate 179 (IVS) had the highest level of expression of *lytA*. In contrast to serotype 6A, all genes were expressed at the highest level in the LVS serotype 6B isolate, save for the *hylA* gene which was expressed at the highest level in the HVS isolate (S78). For serotype 6C, the expression levels of *ply*, *lytA*, *psaA*, and *hylA* genes were higher in the HVS strain (S119) than in the LVS strain (S227), whereas the expression levels of *nanA* and *pspA* were higher in the LVS strain (S227) than in the HVS strain (S119).

Overall, we found great variation in the expression of these virulence genes in different serotypes and even among different strains within the same serotypes. In general, capsular, and non-capsular properties together determined the virulence of *S. pneumoniae* strains. The regulation of virulence levels is complex and elaborate, determined by a variety of virulence genes.

## Discussion

The 300 *S. pneumoniae* isolates studied were isolated from 27 teaching hospitals in China, all from sterile body sites, including blood, cerebrospinal fluid, and pleural effusion, which can result in severe IPD. IPD is an aggressive disease with an annual incidence of 6.6–14 per 100,000 people, and a mortality rate of 10–30% (Sousa et al., 2018). Given that *S. pneumoniae* has become a major challenge to public health systems worldwide, a comprehensive understanding of its clinical characteristics is of great importance.

It has been reported that the distribution of *S. pneumoniae* serotypes exhibits association of specific serotypes with geographical locale, attack rate, age, tendency to cause outbreaks, and propensity to acquire antimicrobial resistance genes (Jourdain et al., 2011). Furthermore, the introduction of vaccines covering specific serotypes has been an effective way to prevent IPD caused by *S. pneumoniae*. The five most common serotypes in this study were: 23F (43, 14.3%), 19A (41, 13.7%), 19F (41, 13.7%), 3 (31, 10.3%), and 14 (27, 9.0%). The serotype coverage rates of PCV7, PCV10, PCV13, and PPV23 vaccines on our bacterial collection was 42.3, 45.3, 73.3 and 79.3%, respectively. The distribution of serotypes and vaccine coverage in our study was similar between the children and adult groups.

Based on reports from the Canadian Public Health Laboratory Network (Demczuk et al., 2013), the predominant *S. pneumoniae* serotypes causing IPD in Canada during the years 2010–2012 were 19A, 7F, 3, and 22F, with PCV13 vaccine coverage ranging from 41 to 66%. Among Turkish children, the most common serotypes causing IPD in 2015–2018 were 19F, 1, and 3, with PCV13 serotypes accounting for 56.2%, as reported by Mehmet Ceyhan et al. (Ceyhan et al., 2020). In Japan, an Asian neighbor of China, the most common serotypes in a study that included 177 patients with IPD from September 2016 to April 2018, were 12F, 3, 23, 19F, 10A, 6C, and 22F, while PCV7, PCV13, and PCV23 had 2.8, 28.2 and 61.0% coverage, respectively (Yanagihara et al., 2021). These findings demonstrate that the distribution of *S. pneumoniae* serotypes and vaccine coverage varies by country, time of isolation, age, *etc*., which may be related to differences in antibiotic use, health conditions, economic situation, and time of vaccine introduction in various regions.

A study from two tertiary children’s hospitals in Beijing (2012–2017) showed that the dominant *S. pneumoniae* serotypes in IPD cases in children were 19F, 19A, 14, 23F, and 6B, with PCV13 having a coverage of 90.1% of the strains (Shi et al., 2019). Likewise, in another study carried out at a hospital in Shenyang, a city in northeast China, from 2010 to 2014, the most common *S. pneumoniae* serotypes among isolates from pediatric IPD cases were 19A, 14, 19F, 23F, and 6B, and the coverage of PCV7, PCV10 and PCV13 vaccines was 53.9, 56.3 and 93.8%, respectively (Zhou et al., 2016). The prevalent serotypes in the two studies mentioned above are similar to ours, but with slightly higher vaccine coverage, which may be due to differences in geographical distribution of the serotypes (Zhou et al., 2016).

PCV7 was introduced in China in 2008, while PCV13 was approved in 2016 and PPV23 was only available for children older than 2 years (Shi et al., 2019). However, vaccination rates of the above-mentioned vaccines are relatively low in most regions of China due to the prohibitive high cost. Hence, many children and elderly people are still vulnerable to the lethal threats of IPD caused by *S. pneumoniae*. Moreover, based on our research, it is worth noting that the serotype coverage of PCV13 has increased significantly relative to PCV7 and PCV10, but not significantly different from that of PPV23. This may be attributed to the continuous rise in the prevalence of PCV13-associated serotypes 19A, 6A, and 3. On this basis, we recommend vaccinating the population in China with PCV13 or PPV23 to reduce the incidence of IPD.

Although the introduction of vaccines has greatly reduced the incidence of IPD for vaccine-covered serotypes, on the other hand, the incidence of IPD caused by non-vaccine-covered serotypes has increased, a phenomenon commonly referred to as “serological replacement”. Data from the Danish National Serological Institute from 1999–2014 showed that the incidence of IPD caused by non-vaccine covered serotypes 8, 9N, 11A, 12F, 15A, 20, 22F, 23B, 24F, and 33F, increased for all ages after the introduction of PCV13 vaccine, compared to the pre-introduction period (Slotved et al., 2016). A review showed that the most common serotypes in European countries before the introduction of the PCV7 vaccine were 14, 6B, 19F, and 23, but after the introduction of this vaccine, the dominant serotypes switched to 1, 19A, 3, 6A, and 7F, which were not covered by PCV7 (Isaacman et al., 2010). In our study, serological replacement was observed for both PCV13 and PPV23 vaccines, although the distribution of prevalent serotypes varied from 2010 to 2015. Therefore, continuous monitoring of the distribution of non-vaccine covered serotypes is essential to assess the effectiveness of vaccine preventative efforts and to effectively control IPD.

Multi-locus sequence typing (MLST) analysis revealed a considerable diversity among the strains, with the most common STs being ST320, ST81, ST271, ST876, and ST3173. Phylogenetic tree analysis revealed that CC320, CC271, CC81, CC876, and CC1263 were the most common clonal complexes. This was consistent with previous studies in which CC320 and CC271 were prevalent complex groups in China (Li et al., 2019). A previous study in Catalonia, Spain, during the year 2009, which consisted of 614 IPD cases, showed that the most common clonal lineages were ST306, CC191, CC230, CC156, ST304, CC1223, and CC180 (Muñoz-Almagro et al., 2011). However, in Singapore, an Asian country, the most common STs causing IPD in children were ST9, ST156, ST236, and ST90 (Jefferies et al., 2011). Therefore, the clonal lineage of *S. pneumoniae* may vary by geographical location.

We observed that the decline in the prevalence of ST320 and ST81 (from 2010–2015) in our study was accompanied by an increase in the prevalence of ST271 and ST876. Given the relationship between serotypes and STs, serotype 19A is dominated by ST320 (80.5%). *S. pneumoniae* strain 19A ST320, derived from an international Taiwan (19F)-14 (ST236) clone, has been reported to be more competitive than its ancestral clone (Hsieh et al., 2013; Chen et al., 2019). Meanwhile, in the current study, serotype 19A ST320 had a higher rate of drug resistance than other STs, and is often associated with multidrug resistance in Asian countries (Shin et al., 2011). Despite a gradual decline (2011–2015) in the isolation rate of serotype 19A ST320 in our study, possibly owing to vaccine pressure, it remained the predominant ST in 2015. In view of its notoriety, the dissemination and sequence type switching of serotype 19A still requires continuous monitoring in China and elsewhere.

In the present study, ST81 was mainly associated with serotype 23F, accounting for 63% of this serotype. The *S. pneumoniae* 23F ST81 lineage was one of the first pandemic clones identified, and was implicated in nearly 40% of penicillin-resistant pneumococcal infections in the United States in the late 1990s (Muñoz et al., 1991). Since then, a study from Beijing raised an alarm that serotype 23F ST81 was non-susceptible to β-lactam antibiotics and was on the rise (Ma et al., 2013). In the present study, although the isolation rate of 23F ST81 went from 14.7% in 2010 to 4% in 2015, the isolation rate in 2014 was still relatively high (10.8%). The discrepancy may be explained by variation in the number of isolates between the years or by a shift in the ST under vaccine pressure. Hence, the distribution of serotype 23F ST81 deserves continuous attention.

ST271 and ST876 were correlated with serotypes 19F and 14, respectively, and were in a rising trend (2010–2015). A previous study demonstrated a worrying rising trend in the 19F CC271 lineage among *S. pneumoniae* strains from acute respiratory infections in Chinese children, from 14.3% in 1997–1998 to 92% in 2010, and was responsible for the increased non-susceptibility rate to β-lactam antibiotics of serotype 19F (Li et al., 2013). Although serotype 19F has been included in the PCV7 vaccine, it presented huge challenges in its manipulation to elicit a satisfactory protective immune response in the human host as it is more resistant to C3 deposition and less sensitive to opsono-phagocytosis (Melin et al., 2009; Poolman et al., 2011). In agreement with this, the isolation rate of serotype 19F in the present study increased from 11.8% in 2010 to 16% in 2015. Thus, trends in serotype 19F and ST271 distribution need to be monitored continuously in the future, especially after the vaccine becomes more widely available.

The isolation rate for ST876 in the present study was relatively low, although it increased alarmingly from 2.9% in 2010 to 10.8% in 2014. Previous studies reported very dramatic increases in the detection rate of *S. pneumoniae* serotype 14 CC876 lineage, increasing notably from 0% in 1997–2000 to 96.4% in 2010 in children with acute respiratory infections in China, completely replacing the previously dominant clonal group CC875 (from 84.2 to 0%) and exhibiting a high level of non-susceptibility rates to β-lactam antibiotics (He et al., 2015). While serotype 14-ST876 did not currently dominate in this study, the increasing isolation rate, and the potent clonal expansiveness of ST876 still sounded alarm bells.

Interestingly, 10 serotypes (12F, 28F, 28A, 4, 25F, 22F, 17, 2, 17A, and 18C) matched only one ST each, implying that the non-vaccine covered serotypes may have a lower clonal diversity. As discussed above, the numbers of non-vaccine covered serotypes are growing and therefore further molecular typing studies of the non-vaccine covered serotypes are needed in the future to assess the effects of vaccines on the epidemiology of *S. pneumoniae.* In addition to this, seven groups of internationally resistance clones such as Spain23F-1, were detected in the present study by sequence alignment with those on the PMEN website. It can be assumed that the increasing international interactions have led to the widespread dissemination of these resistant clones worldwide.

The use of antibiotics is the mainstay of IPD treatment, so it is particularly important to choose effective antimicrobial drugs. All strains in the present study were susceptible to ertapenem, levofloxacin, moxifloxacin, linezolid, and vancomycin, which is in line with most studies in China and elsewhere (Xue et al., 2010; Cai et al., 2018; Golden et al., 2019). These antimicrobial agents may be a valuable reference for empirical medication in IPD treatment.

Penicillin has been recognized as an important choice for the treatment of invasive *S. pneumoniae* infections, but the rising rate of resistance among strains in recent years has sparked concern. In our study, we found that the proportion of PNSP based on the oral breakpoint was 67.7%, compared to 67.7 and 4.3% based on the meningitis and non-meningitis breakpoints, respectively. An analysis involving 1517 invasive *S. pneumoniae* strains from children in Germany between 1997 and 2004, showed that only 5.1 and 1.1% of the isolates were penicillin-intermediate and resistant, respectively (Reinert et al., 2007).

In China’s bordering country, Cambodia, 46% of *S. pneumoniae* strains from children with invasive infections (2007–2012) were PNSP based on the meningitis breakpoint (Moore et al., 2016). The proportion of PNSP in our study was significantly higher than those in the two studies mentioned above, which may be explained by variation in the population included and the misuse of these over-the-counter antibiotics in China due to their availability and convenience from numerous licensed and non-licensed sources (Troy, 1992). Furthermore, the present study included isolates from both adults and children whereas the previous studies only focused on the pediatric population. In general, the adult population tends to be exposed to antibiotics more than children, resulting in an increased overall non-susceptibility rate.

Notably, most of the studied isolates (over 95%) were resistant to macrolides (azithromycin, clarithromycin, erythromycin) and clindamycin. This may be related to the frequent clinical use of these antibiotics during the past years, and imply that these antibiotics should no longer be recommended for treating *S. pneumoniae* infections. The widespread clinical use of macrolide antibiotics was strongly associated with the rise in resistance, and the major resistance mechanism is the acquisition of the *erm*(B) gene encoding a 23S methylase or the *mef* gene encoding an active (proton dependent) efflux pump (Cornick and Bentley, 2012). Besides, the high resistance rate of *S. pneumoniae* to clindamycin is noteworthy and further studies are needed to elucidate the exact resistance mechanism involved.

In terms of the distribution of antibiotic drug resistance across serotypes, serotypes 23F, 19F, 19A, and 14, exhibited higher resistance rates, while serotype 3 exhibited a relatively lower resistance rate. As previously mentioned, high levels of resistance in serotypes 23F, 19F, 19A, and 14 may be associated with the widespread international spread of ST81, ST271, ST320, and ST876, respectively. As for serotype 3, a similar resistance pattern was observed in Mexico, where 196 *S. pneumoniae* strains were susceptible to almost all the antibiotics tested, the predominant clonal complex group being CC180 (71.4%) (Echániz-Aviles et al., 2019). In our study, serotype 3 had a highly clonal diversity, dominated by ST505 (19.4%) and ST180 (19.4%). By whole genome sequencing of 616 strains of serotype 3 from England and Wales over a 15-year period, Groves et al. found that the composition of their clade changed along with shifts in antibiotic resistance (Groves et al., 2019). Thus, the clonal lineage and resistance pattern of serotype 3 should be closely monitored.

Another noticeable finding in this study was that the resistance rates of vaccine-covered serotypes were generally higher than those of non-vaccine-covered serotypes, which is in agreement with a previous report (Zhao et al., 2020). With the increasing number of non-vaccines covered serotypes causing IPD, the resistance levels of these serotypes should be carefully monitored under the dual pressure of antibiotics and vaccines.

An analysis of the annual antibiotic resistance rates showed a decrease from 2010 to 2015 for penicillin, cefuroxime, ceftriaxone, cefepime, cefaclor and trimethoprim/sulfamethoxazole. Regarding penicillin, we speculated that this phenomenon may be related to the penicillin breakpoint update in 2008 and the introduction of the PCV7 vaccine. Karlowsky et al. (2018) reported that 6001 *S. pneumoniae* isolates from Canada showed a small increase (*P* < 0.05) in susceptibility to penicillin and ceftriaxone during the period 2011–2015. In our study, the increased susceptibility of *S. pneumoniae* to cephalosporin antibiotics may be due to a decrease in serotypes 19A and 23F, which were resistant to a variety of antibiotics, including cephalosporins. Furthermore, a 20-year continuous global monitoring by the SENTRY Antimicrobial Surveillance Program (1997–2016) found that the susceptibility rates of ceftriaxone, erythromycin, clindamycin, tetracycline, and trimethoprim-sulfamethoxazole decreased in the first 12–14 years, and increased thereafter in the last 6–8 years (Sader et al., 2019). From a long-term point of view, a global collaboration with large samples and a prolonged time scale will help elucidate the current and changing trends of drug resistance to combat invasive infections caused by *S. pneumoniae* effectively.

Virulence is a key indicator for evaluating pathogenicity. We performed *in vivo* animal experiments to assess the virulence levels of the prevalent serotypes using the intraperitoneal infection model. Our study showed that serotypes 23F, 19A, 19F, and 14 strains were the prevalent avirulent serotypes while serotype 3 exhibited high virulence levels. However, the virulence levels of strains in serogroup 6 varied among serotypes and strains. A previous meta-analysis consisting of nine studies showed that the outcome of IPD was strongly associated with the serotype (Weinberger et al., 2010). In that study, serotypes 1, 7F, and 8 were associated with decreased risk ratio (RR) while serotypes 3, 6A, 6B, 9N, and 19F, were associated with increased relative risk (RR). Another study (Briles et al., 1992) demonstrated the strong association between capsular serotypes and virulence of the *S. pneumoniae* strains through *in vivo* virulence experiments in mice. Based on their observation, all serotype 4 strains, 40% of serotype 3 and 60% of serogroup 6, were virulent for mice, whilst strains of serogroups/types 14, 19, and 23, were avirulent. This was partly in line with our findings. Furthermore, according to our investigations, all serotype 3 strains from various clonal origins were virulent while all strains of serotypes 23F, 19F, 19A, and 14 from diverse clonal origins were avirulent, suggesting that the different capsular polysaccharides conferred pneumococci with intrinsically different virulence properties. Intriguingly, we found that strains of serotype 3 were characterized by high virulence and low resistance, while strains of serotypes 23F, 19F, 19A, and 14 were of low virulence and high resistance. Our findings are similar to those of Azoulay-Dupuis et al. (2000). In addition, a case-control study also showed that the clinical presentation of adult pneumonia caused by PNSP was milder than that of PSSP, suggesting that it is possible that resistance “carries a cost” (Einarsson et al., 1998). We speculated that strains of low virulent serotypes may be more prone to long-term colonization to acquire resistance through exposure to more antibiotics.

The virulence level of strains in serogroup 6 as per our mouse sepsis model was considerably complicated and complex, with great variation observed in the virulence levels of different strains within the same serotype (6A, 6B or 6C). Previous research (Briles et al., 1992) has shown that only 60% of strains in serogroup 6 were virulent for mice, which reinforced the heterogeneity of the virulence of serogroup 6 pneumococci. The fact that strains with the same serotype exhibit different virulence levels suggest presence of virulence determinants other than capsular polysaccharides. It is also worth noting that several studies (Polissi et al., 1998; Lau et al., 2001) using signature-tagged mutagenesis have unveiled a large set of genes that can influence the virulence of *S. pneumoniae*. Numerous non-capsular virulence genes, including *ply*, *lytA*, *nanA*, *psaA*, *pspA* and *hylA*, have been shown to be involved in the regulation of pneumococcal virulence (Ogunniyi et al., 2002; LeMessurier et al., 2006). *Ply* encoding pneumolysin, which can contribute to the early development of IPD by facilitating the invasion of *S. pneumoniae* from the lung into the bloodstream. Significantly, pneumolysin is a multifunctional toxin exhibiting both hemolytic and complement activities, and its deficiency would lead to a reduction in the virulence of the *S. pneumoniae* strain (Rubins et al., 1996). *LytA*, encoding an amidase with autolytic activity, exerts its virulence by releasing pneumolysin and inflammatory peptidoglycan from lysing bacteria (Kadioglu et al., 2008). It was reported that *LytA*-negative *S. pneumoniae* exhibited a lower virulence level through murine models of pneumonia and bacteremia (Canvin et al., 1995). All *S. pneumoniae* strains express *nanA* which encoding neuraminidase, it is essential for promoting *S. pneumoniae* adhesion to and invasion of uman brain microvascular endothelial cells (Uchiyama et al., 2009). *PsaA* is thought to encode a pneumococcal adhesin. In murine models of pneumonia, bacteremia and colonization, knockout of *psaA* abolished the virulence of *S. pneumoniae* (Marra et al., 2002). *PspA* impairs the fixation of complement component C3 on the surface of pneumococcal cells, thereby inhibiting complement-mediated opsonization. In addition, *pspA* encodes a lactoferrin-binding protein that protects bacteria from being killed by apolactoferrin (Kadioglu et al., 2008). *HylA* encodes the hyaluronate lyase enzyme which degrades hyaluronan, then destroying the structure of the connective tissue and exposing it to endo- and exogenous factors, including various bacterial toxins (Jedrzejas et al., 2002).

Therefore, the expression level of above genes amongst serogroup 6 strains was measured and compared. Our results revealed that the expression of virulence genes varied among these strains, but did not correspond significantly to the virulence phenotype of the *in vivo* experiments. This prompted us to think that pneumococcal virulence may be collaboratively regulated by multiple virulence genes, and that this regulation is highly complex and multifactorial. Robinson et al. (2002) identified the major clones of 212 *S. pneumoniae* strains from 39 countries by MLST, and the results indicated that the virulent serogroup 6 strains originated from the avirulent ancestral clones and went through multiple evolution to become virulent. It is possible that horizontal recombination or evolution of virulence genes between strains contributed to a diversity in virulence. Overall, our study suggests that capsular polysaccharide and non-capsular virulence genes are collectively responsible for the virulence diversity of *S. pneumoniae* strains.

This study has several limitations. Firstly, the strains collected in this study were from different time periods, which could lead to bias in the results. Secondly, the strains were mainly from tertiary teaching hospitals in China; samples from remote and rural areas were not included. Thirdly, the virulence of some of the rarer serotypes and non-vaccine covered serotypes were not evaluated.

## Conclusion

Overall, our study provides a comprehensive insight into the epidemiological and virulence diversity of *S. pneumoniae* strains causing IPD in China. The most common serotypes in this study were 23F, 19A, 19F, 3, and 14. The serotype coverages of PCV7, PCV10, PCV13, and PPV23 vaccines on the bacterial collection were 42.3, 45.3, 73.3 and 79.3%, respectively. The most common STs were ST320, ST81, ST271, ST876, and ST3173. All strains were susceptible to ertapenem, levofloxacin, moxifloxacin, linezolid, and vancomycin, but highly resistant to macrolides and clindamycin (>95%). Serotype 3 strains were characterized by high virulence levels and low antimicrobial-resistance rates, while strains of serotypes 23F, 19F, 19A, and 14, exhibited low virulence and high resistance rates to antibiotics.

## Data Availability Statement

The original contributions presented in the study are included in the article/Supplementary Material, further inquiries can be directed to the corresponding author/s.

## Ethics Statement

The animal study was reviewed and approved by the Medical Ethics Committee of Peking Union Medical College Hospital (No. S-263).

## Author Contributions

ZL, HZ, YX, and JZ conceived and designed the work. MZ, HA, CQ, BJ, and YW performed the experiments. MZ, ZW, LZ, and TK performed the data analysis and wrote the manuscript. All authors read and approved the final manuscript.

## Conflict of Interest

The authors declare that the research was conducted in the absence of any commercial or financial relationships that could be construed as a potential conflict of interest.

## Publisher’s Note

All claims expressed in this article are solely those of the authors and do not necessarily represent those of their affiliated organizations, or those of the publisher, the editors and the reviewers. Any product that may be evaluated in this article, or claim that may be made by its manufacturer, is not guaranteed or endorsed by the publisher.

## Footnotes

1
https://pubmlst.org
