# Supplementary material for: Serotype Distribution, Antimicrobial Susceptibility, Multilocus Sequencing Type and Virulence of Invasive Streptococcus pneumoniae in China: A Six-Year Multicenter Study
Source: Front Microbiol. 2022 Jan 13;12:798750. doi: 10.3389/fmicb.2021.798750 (PMC8793633; doi:10.3389/fmicb.2021.798750)
Supplement: Supplementary file 1 [file Data_Sheet_1.docx]

**Table S1 Primers for MLST and RT-PCR**

| **Gene** | **Primer** | **Sequence (5'→3')** |
| --- | --- | --- |
| *aroE* | *aroE* F | GCCTTT GAGGCG ACAGC |
|  | *aroE* R | TGCAGT TCA (G/A) AA ACAT (A/T) TTCTAA |
| *gdh* | *gdh* F | ATGGACAAACCAGC (G/A/T/C) AG (C/T) TT |
|  | *gdh* R | GCTTGAGGTCCCAT (G/A) CT (G/A/T/C) CC |
| *gki* | *gki* F | GGCATTGGAATG GGATCACC |
|  | *gki* R | TCTCCCGCAGCTGACAC |
| *recP* | *recP* F | GCCAACTCAGGTCATCCAGG |
|  | *recP* R | TGCAACCGTAGCATTGTAAC |
| *spi* | *spi* F | TTATTCCTCCTGATTCTGTC |
|  | *spi* R | GTGATTGGCCAGAAGCGGAA |
| *xpt* | *xpt* F | TTATTAGAAGAGCGCATCCT |
|  | *xpt* R | AGATCTGCCTCCTTA AATAC |
| *ddl* | *ddl* F | TGC (C/T) CAAGTTCCTTATGTGG |
|  | *ddl* R | CACTGGGT(G/A) AAA CC(A/T) GGCAT |
| 16S rRNA | 16S rRNA F | GTAGTCCACGCTGAAACGATGATG |
|  | 16S rRNA R | CTGTCCCGAAGGAAAACTCTATCT |
| *nanA* | *nanA* F | CCAGCCTATAGCGACAAGGG |
|  | *nanA* R | TGTTCCAGGACCTACACCCA |
| *lytA* | *lytA* F | AAATGGGGCATTAGCCGTGA |
|  | *lytA* R | AGTACCAGTTGCCGTCTGTG |
| *ply* | *ply* F | AGGCTTGGGACAGAAATGGG |
|  | *ply* R | GAAATCGTCCGCTTACGCAC |
| *psaA* | *psaA* F | ACCAGCGAAGGAGCATTCAA |
|  | *psaA* R | TGGACGGTCATCCACACTTG |
| *pspA* | *pspA* F | GTGCTCCTCTTCAATCTAAAT |
|  | *pspA* R | CAGCTTGCTCACCGTCTGAGT |
| *HylA* | *HylA* F | TTCTACCATTCGCTCGATTGA |
|  | *HylA* R | AGCACCCGTATAGGCAACATT |

**Table S2 Serotype distribution of 300 invasive *S. pneumoniae* isolates involved in this study**

| **Serotype** | **No. (%) of isolates** | **PPV** | **PCV** | **Serotype** | **No. (%) of isolates** | **PPV** | **PCV** |
| --- | --- | --- | --- | --- | --- | --- | --- |
| 23F | 43 (14.3) | PPV23 | PCV7 | 28F | 3 (1.0) | NA | NA |
| 19A | 41 (13.7) | PPV23 | PCV13 | 9V | 3 (1.0) | PPV23 | PCV7 |
| 19F | 41 (13.7) | PPV23 | PCV7 | 5 | 2 (0.7) | PPV23 | PCV10 |
| 3 | 31 (10.3) | PPV23 | PCV13 | 15F | 2 (0.7) | NA | NA |
| 14 | 27 (9.0) | PPV23 | PCV7 | 25A | 2 (0.7) | NA | NA |
| 6A | 12 (4.0) | NA | PCV13 | 28A | 2 (0.7) | NA | NA |
| 6B | 11 (3.7) | PPV23 | PCV7 | 33B | 2 (0.7) | NA | NA |
| 20 | 7 (2.3) | PPV23 | NA | 6C | 2 (0.7) | NA | NA |
| 34 | 7 (2.3) | NA | NA | 7C | 2 (0.7) | NA | NA |
| 15C | 6 (2.0) | NA | NA | 7F | 2 (0.7) | PPV23 | PCV10 |
| 1 | 5 (1.7) | PPV23 | PCV10 | 9A | 2 (0.7) | NA | NA |
| 8 | 4 (1.3) | PPV23 | NA | 9N | 2 (0.7) | PPV23 | NA |
| 11A | 4 (1.3) | PPV23 | NA | 2 | 1 (0.3) | PPV23 | NA |
| 12F | 4 (1.3) | PPV23 | NA | 4 | 1 (0.3) | PPV23 | PCV7 |
| 15B | 4 (1.3) | PPV23 | NA | 17 | 1 (0.3) | NA | NA |
| 23A | 4 (1.3) | NA | NA | 17A | 1 (0.3) | NA | NA |
| 13 | 3 (1.0) | NA | NA | 18C | 1 (0.3) | PPV23 | PCV7 |
| 29 | 3 (1.0) | NA | NA | 22F | 1 (0.3) | PPV23 | NA |
| 10A | 3 (1.0) | PPV23 | NA | 25F | 1 (0.3) | NA | NA |
| 15A | 3 (1.0) | NA | NA | NT | 1 (0.3) | NA | NA |
| 24F | 3 (1.0) | NA | NA |  |  |  |  |

NA, not available

**Tabel S3 MLST distribution of 300 invasive *S. pneumoniae* isolates involved in this study**

| **Sequence Type** | **No. of isolates** | **Sequence Type** | **No. of isolates** | **Sequence Type** | **No. of isolates** |
| --- | --- | --- | --- | --- | --- |
| 320 | 34(11.3%) | 3263 | 1(0.3%) | 10101 | 1(0.3%) |
| 81 | 28(9.3%) | 12904 | 1(0.3%) | 143 | 1(0.3%) |
| 271 | 26(8.7%) | 855 | 1(0.3%) | 11952 | 1(0.3%) |
| 876 | 18(6.0%) | 9237 | 1(0.3%) | 15 | 1(0.3%) |
| 3173 | 8(2.7%) | 872 | 1(0.3%) | 12224 | 1(0.3%) |
| 180 | 6(2.0%) | 2572 | 1(0.3%) | 6935 | 1(0.3%) |
| 505 | 6(2.0%) | 230 | 1(0.3%) | 2758 | 1(0.3%) |
| 3397 | 5(1.7%) | 83 | 1(0.3%) | 386 | 1(0.3%) |
| 2296 | 4(1.3%) | 3545 | 1(0.3%) | 12903 | 1(0.3%) |
| 4389 | 4(1.3%) | 12908 | 1(0.3%) | 6946 | 1(0.3%) |
| 90 | 4(1.3%) | 3590 | 1(0.3%) | 12905 | 1(0.3%) |
| 236 | 4(1.3%) | 12916 | 1(0.3%) | 6993 | 1(0.3%) |
| 14726 | 4(1.3%) | 105 | 1(0.3%) | 12907 | 1(0.3%) |
| 4655 | 4(1.3%) | 9240 | 1(0.3%) | 7388 | 1(0.3%) |
| 9114 | 4(1.3%) | 902 | 1(0.3%) | 12909 | 1(0.3%) |
| 6945 | 4(1.3%) | 9396 | 1(0.3%) | 7402 | 1(0.3%) |
| 12902 | 3(1.0%) | 4467 | 1(0.3%) | 12911 | 1(0.3%) |
| 3398 | 3(1.0%) | 10086 | 1(0.3%) | 7725 | 1(0.3%) |
| 4660 | 3(1.0%) | 983 | 1(0.3%) | 12913 | 1(0.3%) |
| 342 | 3(1.0%) | 11967 | 1(0.3%) | 408 | 1(0.3%) |
| 4560 | 3(1.0%) | 4640 | 1(0.3%) | 12915 | 1(0.3%) |
| 1263 | 3(1.0%) | 554 | 1(0.3%) | 9230 | 1(0.3%) |
| 1937 | 3(1.0%) | 338 | 1(0.3%) | 12917 | 1(0.3%) |
| 2754 | 3(1.0%) | 12906 | 1(0.3%) | 9231 | 1(0.3%) |
| 280 | 2(0.7%) | 1464 | 1(0.3%) | 673 | 1(0.3%) |
| 12449 | 2(0.7%) | 12910 | 1(0.3%) | 9234 | 1(0.3%) |
| 3500 | 2(0.7%) | 1504 | 1(0.3%) | 13199 | 1(0.3%) |
| 242 | 2(0.7%) | 12914 | 1(0.3%) | 5872 | 1(0.3%) |
| 4216 | 2(0.7%) | 12919 | 1(0.3%) | 14346 | 1(0.3%) |
| 880 | 2(0.7%) | 12918 | 1(0.3%) | 5893 | 1(0.3%) |
| 2248 | 2(0.7%) | 12920 | 1(0.3%) | 14348 | 1(0.3%) |
| 99 | 2(0.7%) | 9238 | 1(0.3%) | 6011 | 1(0.3%) |
| 9803 | 2(0.7%) | 13200 | 1(0.3%) | 14350 | 1(0.3%) |
| 3176 | 2(0.7%) | 9242 | 1(0.3%) | 6202 | 1(0.3%) |
| 12901 | 2(0.7%) | 1876 | 1(0.3%) | 14705 | 1(0.3%) |
| 4745 | 2(0.7%) | 9244 | 1(0.3%) | 6227 | 1(0.3%) |
| 14347 | 2(0.7%) | 14349 | 1(0.3%) | 14707 | 1(0.3%) |
| 5244 | 2(0.7%) | 9785 | 1(0.3%) | 6327 | 1(0.3%) |
| 12912 | 1(0.3%) | 14351 | 1(0.3%) | 14727 | 1(0.3%) |
| 10106 | 1(0.3%) | 10085 | 1(0.3%) | 6429 | 1(0.3%) |
| 9243 | 1(0.3%) | 14706 | 1(0.3%) | 6541 | 1(0.3%) |

**Table S4 Sequence type and serotype of seven PMEN clones**

| **PMEN clones** | **ST** | **Serotype** | **No. of isolates** | **Serological replacement** |
| --- | --- | --- | --- | --- |
| **Spain^23F^-1** | **ST81** | **19F** | **1** | **Yes** |
|  | ST81 | 23F | 27 | No |
| Netherlands^3^-31 | ST180 | 3 | 6 | No |
| Taiwan^19F^-14 | ST236 | 19F | 4 | No |
| Spain^6B^-2 | ST90 | 6B | 4 | No |
| Taiwan^23F^-15 | ST242 | 23F | 2 | No |
| **Denmark14-32** | **ST230** | **23A** | **1** | **Yse** |
| **Colombia^23F^-26** | **ST338** | **23A** | **1** | **Yes** |

**Table S5 Comparison of antimicrobial susceptibility results of the 300 *S. pneumoniae* isolates within and without vaccine coverage in China**

| **Antibiotics** | **R (%)** | | | | | |
| --- | --- | --- | --- | --- | --- | --- |
|  | **PCV (n=220)** | **Non-PCV (n=80)** | ***P* value** | **PPV23 (n=238)** | **Non-PPV23 (n=62)** | ***P* value** |
| P^a^ | 0 | 0 | NA | 0 | 0 | NA |
| P^b^ | 78.6 | 37.5 | **<0.0001** | 71.4 | 53.2 | **0.01** |
| P^c^ | 60.5 | 1.2 | **<0.0001** | 52.5 | 14.5 | **<0.0001** |
| AMC^a^ | 0.5 | 0 | 0.6553 | 0.4 | 0 | 0.442 |
| CXM^d^ | 74.5 | 20 | **<0.0001** | 67.2 | 32.3 | **<0.0001** |
| CXM^c^ | 78.2 | 26.2 | **<0.0001** | 71 | 38.7 | **<0.0001** |
| CRO^a^ | 10 | 0 | **0.0072** | 9.2 | 0 | **0.0274** |
| CRO^b^ | 34.5 | 1.2 | **<0.0001** | 30.7 | 6.5 | **0.0002** |
| FEP^a^ | 5 | 1.2 | 0.2485 | 4.6 | 1.6 | 0.4767 |
| FEP^b^ | 37.3 | 8.8 | **<0.0001** | 34.5 | 11.3 | **0.0007** |
| CEC | 77.3 | 27.5 | **<0.0001** | 69.7 | 41.9 | **0.0001** |
| ETP | 0 | 0 | NA | 0 | 0 | NA |
| IPM | 3.2 | 5 | 0.6998 | 3.4 | 4.8 | 0.8865 |
| MEM | 3.2 | 1.2 | 0.5853 | 2.9 | 1.6 | 0.9012 |
| LEV | 0 | 0 | NA | 0 | 0 | NA |
| MXF | 0 | 0 | NA | 0 | 0 | NA |
| SXT | 68.2 | 57.5 | 0.113 | 68.9 | 51.6 | **0.0164** |
| DA | 97.3 | 91.2 | **0.0482** | 96.2 | 93.5 | 0.5631 |
| AZM | 96.8 | 93.8 | 0.4012 | 96.2 | 95.2 | 0.9953 |
| CLR | 96.8 | 93.8 | 0.4012 | 96.2 | 95.2 | 0.9953 |
| E | 96.8 | 93.8 | 0.4012 | 96.2 | 95.2 | 0.9953 |
| LZD | 0 | 0 | NA | 0 | 0 | NA |
| VA | 0 | 0 | NA | 0 | 0 | NA |
| C | 90.9 | 93.8 | 0.5702 | 90.8 | 95.2 | 0.3895 |
| TET | 94.5 | 91.2 | 0.443 | 93.3 | 95.2 | 0.7988 |

^a^ non-meningitis breakpoint

^b^ meningitis breakpoint

^c^ oral breakpoint

^d^ injection breakpoint

NA, not available

P, penicillin; AMC, amoxicillin/clavulanic; CXM, cefuroxime; CRO, ceftriaxone; FEP, cefepime; CEC, cefaclor; ETP, ertapenem; IPM, imipenem; MEM, meropenem; LEV, levofloxacin; MXF, moxifloxacin; SXT, trimethoprim/sulfamethoxazole; DA, clindamycin; AZM, azithromycin; CLR, clarithromycin; E, erythromycin; LZD, linezolid; VA, vancomycin; C, chloramphenicol; TET, tetracycline


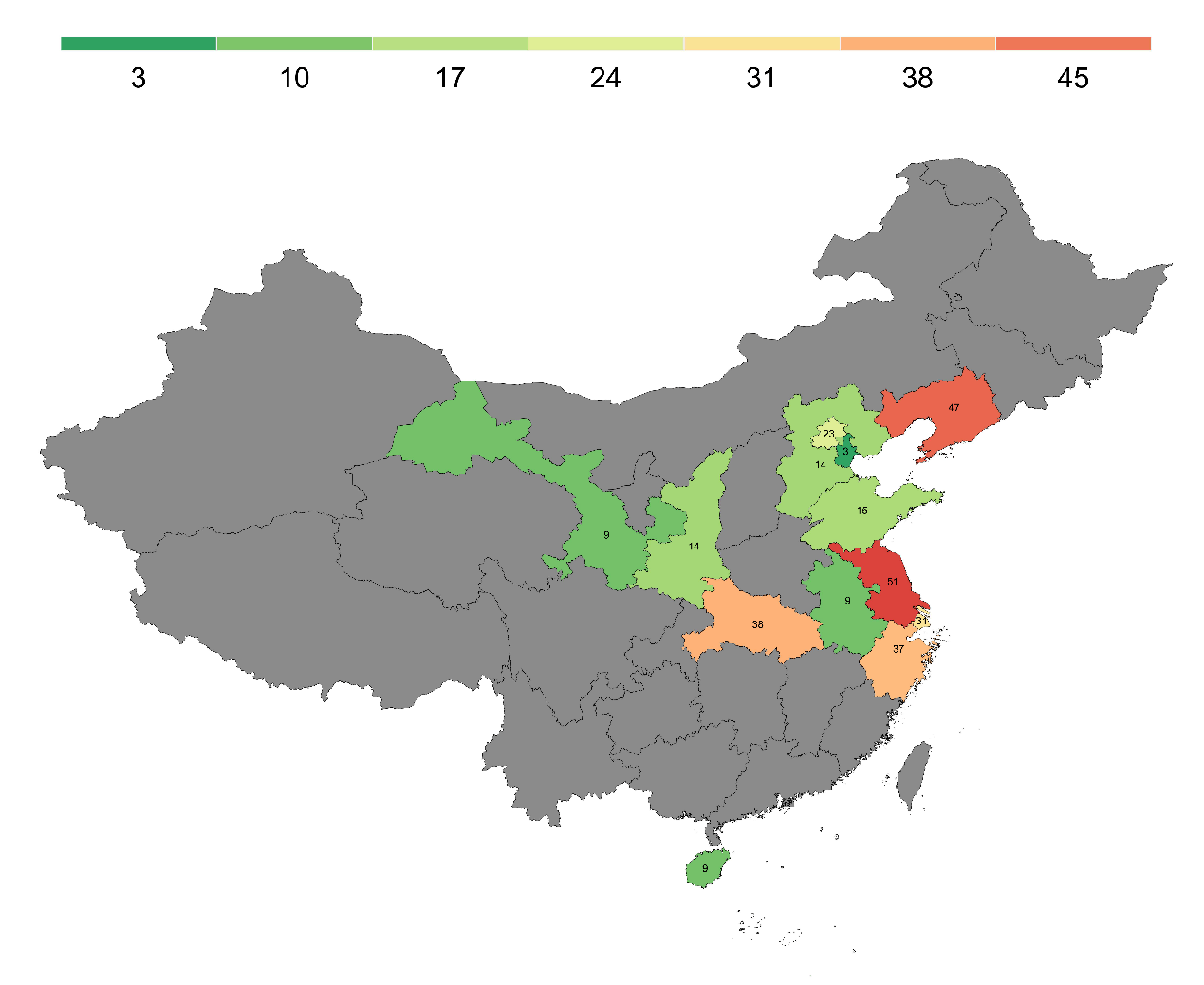


**Fig S1 Geographic distribution of 300 invasive *S. pneumoniae* isolates**


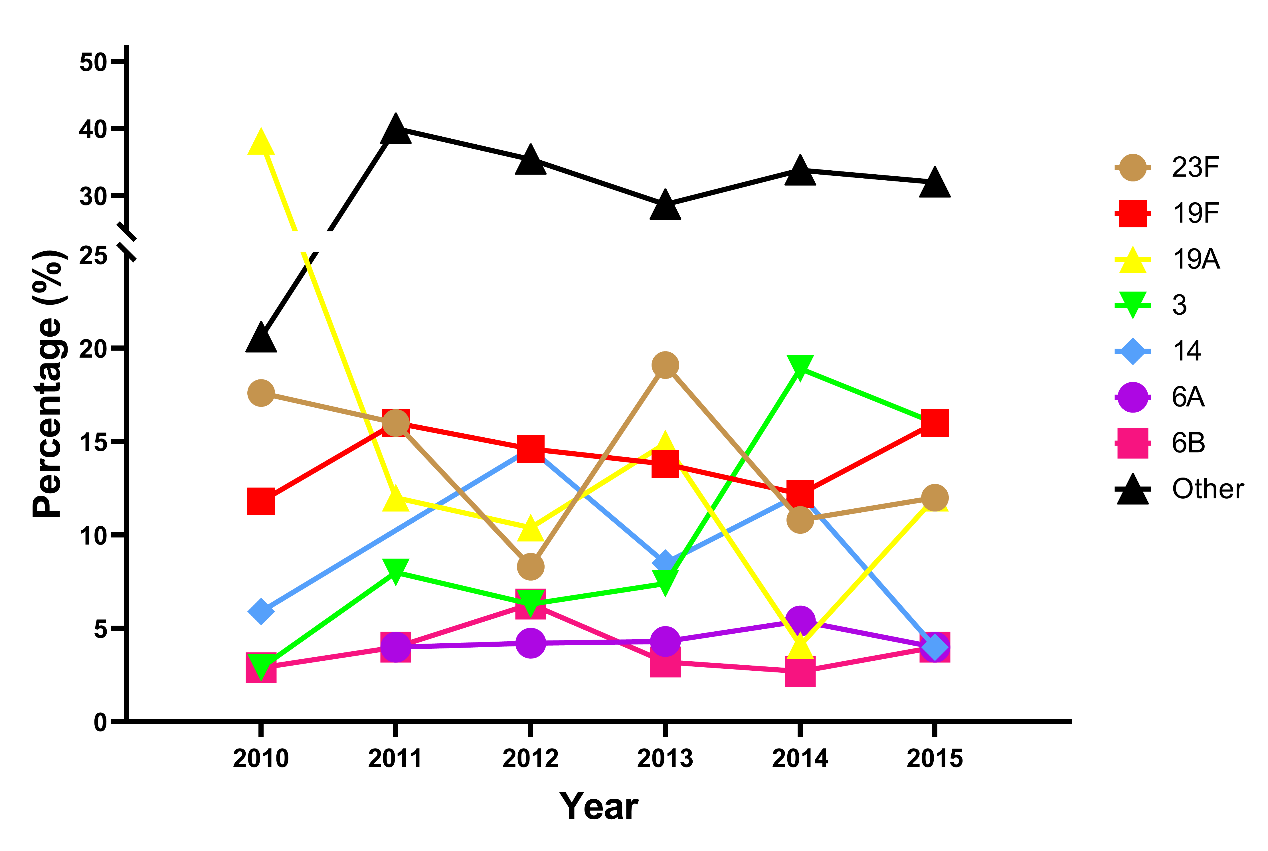


**Fig S2 Serotype distribution of 300 invasive *S. pneumoniae* isolates during the study period 2010-2015**


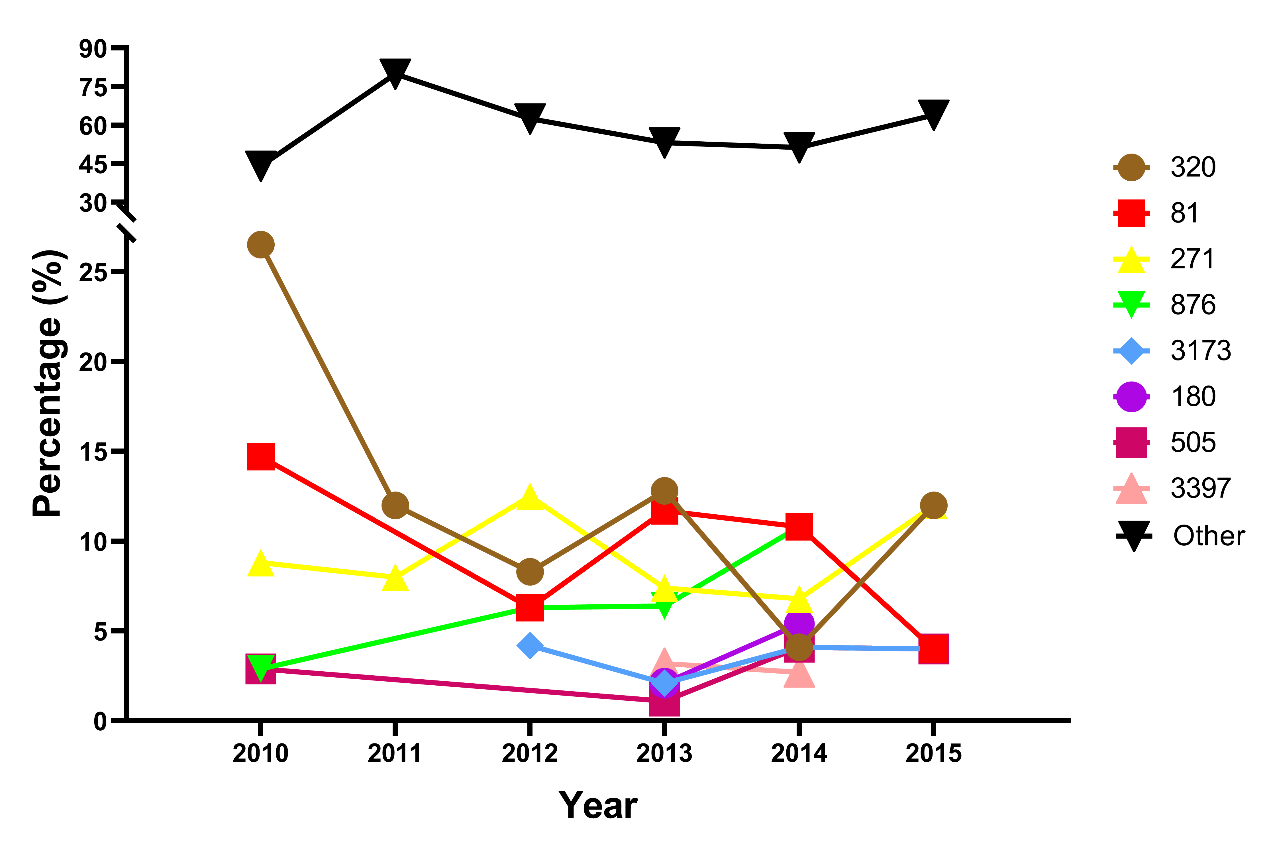


**Fig S3 Sequence type (ST) distribution of 300 invasive *S. pneumoniae* isolates during the study period 2010-2015**
